# ATM Induces Cell Death with Autophagy in Response to H_2_O_2_ Specifically in *Caenorhabditis elegans* Nondividing Cells

**DOI:** 10.1155/2018/3862070

**Published:** 2018-07-02

**Authors:** Takahito Moriwaki, Akira Yamasaki, Qiu-Mei Zhang-Akiyama

**Affiliations:** ^1^Laboratory of Stress Response Biology, Graduate School of Science, Kyoto University, Kitashirakawa-Oiwakecho, Sakyo-ku, Kyoto 606-8502, Japan; ^2^Department of Immunology and Genomic Medicine, Graduate School of Medicine, Kyoto University, Yoshida Konoe-cho, Sakyo-ku, Kyoto 606-8501, Japan

## Abstract

**Introduction:**

Ataxia-telangiectasia-mutated (ATM) kinase is a master regulator of the DNA damage response and is directly activated by reactive oxygen species (ROSs) in addition to DNA double-stranded breaks. However, the physiological function of the response to ROSs is not understood.

**Purpose:**

In the present study, we investigated how ATM responds to ROSs in *Caenorhabditis elegans (C. elegans)*.

**Materials and Methods:**

First, we measured sensitivities of larvae to DNA-damaging agents and ROSs. Next, we analyzed the drug sensitivities of fully matured adult worms, which consist of nondividing somatic cells. Dead cell staining with acridine orange was performed to visualize the dead cells. In addition, we performed GFP reporter assays of *lgg-1*, an autophagy-related gene, to determine the types of cell death.

**Results:**

*atm-1*(*tm5027*) larvae showed a wide range of sensitivities to both DNA-damaging agents and ROSs. In contrast, fully matured adult worms, which consist of nondividing somatic cells, showed sensitivity to DNA-damaging agent, NaHSO_3_, but they showed resistance to H_2_O_2_. Dead cell staining and GFP reporter assays of *lgg-1* suggest that *C. elegans* ATM-1 induces the cell death with autophagy in intestinal cells in response to H_2_O_2_.

**Conclusion:**

We revealed that ATM induces cell death in response to H_2_O_2_.

## 1. Introduction

Ataxia-telangiectasia-mutated (ATM) kinase plays a critical role in the DNA damage response and DNA repair [1]. In response to DNA double-stranded breaks (DSBs), ATM is activated by autophosphorylation of serine 1981 and induces DNA repair, cell cycle arrest, and cell death together with the MRE11-RAD50-NBS1 (MRN) complex [2]. Dysfunction of ATM results in ataxia-telangiectasia (AT) in humans [3]. AT is an autosomal recessive inherited disorder with characteristic symptoms such as the cerebellar ataxia, oculocutaneous telangiectasia, immunodeficiency, and cancer predisposition [3]. Nijmegen breakage syndrome (NBS), which is induced by the dysfunction of NBS1, is also an autosomal recessive inherited disorder with characteristic symptoms, such as immunodeficiency and cancer predisposition similar with AT [3]. Although NBS1 and ATM function in the same pathway, the cerebellar ataxia is not observed in NBS patients [3]. Therefore, ATM is considered to have additional roles to DNA damage response (DDR).

Recently, it was reported that the oxidation of cysteine 2991 of ATM results in the formation of disulfide bond between coupled cysteine 2991 of dimeric ATM followed by autophosphorylation of serine 1981 [4], which phosphorylate p53 or Chk2 in vitro. This suggests that ATM can be directly activated by reactive oxygen species (ROSs) without DSBs [4]. ROSs, such as superoxide anion radical (^•^O_2_^−^), hydrogen peroxide (H_2_O_2_), and hydroxyl radical (^•^OH), are generated by normal cell metabolism, drug treatments, and radiation [5].

As lots of ROSs are generated in the cerebellum, the response of ATM to ROSs is expected to be related to the cerebellar ataxia in AT syndrome [6]. However, the physiological function of the oxidized active dimer of ATM in nondividing cells has not been determined.

In order to elucidate the physiological function of the response of ATM to ROSs in nondividing cells, we analyzed the function of ATM using *Caenorhabditis elegans (C. elegans)*. *C. elegans* has been used as a model organism of aging and apoptosis [7]. In *C. elegans*, the cell fates have been completely determined, and adult hermaphrodites contain 959 nondividing somatic cells [8, 9]. Therefore, *C. elegans* is a good model animal for the analysis of the stress response in nondividing cells. Thus far, we have reported the function of DNA mismatch repair (MMR) unique to nondividing cells using *C. elegans* [10].

In previous studies, *C. elegans* ATM-1 (CeATM-1) was found to protect germ cells from *γ*-rays irradiation, suggesting that CeATM-1 functions in DSB repair as in mammals [11]. In addition, we previously reported that *atm-1*(*tm5027*) worms exhibited sensitivity to methyl methanesulfonate (MMS) at both larval and adult worm stages [10]. This suggests that ATM-1 is also required for DSB repair in both dividing and nondividing somatic cells.

Thus, *C. elegans* was expected as good animal model to investigate the response to ROSs of ATM in the neuron. In this study, we determined the function of ATM in nondividing cells and speculated the cause of the cerebellar ataxia.

## 2. Materials and Methods

### 2.1. *C. elegans* Strains and Culture Conditions

The wild-type strain (Bristol N2) [7], JK2739[*lin-6*(*e1466*) *dpy-5*(*e61*) *I*/*hT2* [*bli-4*(*e937*) *let-?*(*q782*) *qIs48*] (*I;III*)] [12], and MAH236; ([*lgg-1*p::GFP::*lgg-1* + *odr-1*p::RFP]) [13] were supplied by the Caenorhabditis Genetics Center (Minneapolis, USA). The *atm-1*(*tm5027*) mutant was supplied by the National BioResource Project (Tokyo, Japan) [10]. A deletion in the *atm-1* gene was verified by PCR using two primer pairs listed in the Supplementary Table (available here). The *atm-1*(*tm5027*) mutant worms were backcrossed with Bristol N2 twice and maintained with the GFP balancer *hT2* to avoid the accumulation of mutations [12]. The *lgg-1* reporter strain (*tm5027*, [*lgg-1*p::GFP::*lgg-1* + *odr-1*p::RFP]) was generated by crossing each strain. Worms were cultured on 50 mm NGM plates containing 0.3% (*w*/*v*) NaCl, 0.25% (*w*/*v*) polypeptone, 0.005% (*w*/*v*) cholesterol, 1 mM CaCl_2_, 1 mM MgSO_4_, 25 mM potassium phosphate (pH 6.0), and 0.17% (*w*/*v*) agar with a lawn of *Escherichia coli (E. coli)* OP50 at 20°C [14].

### 2.2. Establishment of a Stable *atm-1*(*tm5027*) Worm Line

However, its contribution to somatic cells was not clarified. In order to analyze the function of CeATM-1 in somatic cells, we first established a stable maintenance system for *atm-1*(*tm5027*) worms, because it was previously reported that CeATM-1 contributes to genome integrity in *C. elegans* germ cells [11]. We backcrossed the *atm-1*(*tm5027*) worms with wild-type N2 worms twice, and then we crossed backcrossed *atm-1*(*tm5027*) worms with JK2739 (*hT2*) worms to keep the worms heterozygous [12]. We maintained *atm-1*(*tm5027*/*hT2*) heterozygous worms by picking GFP-positive worms until use and isolated *atm-1*(*tm502*7) homozygous worms for experiments by picking GFP-negative worms. Previous *atm-1* knockdown worms exhibited normal growth [15, 16]. Backcrossed *atm-1*(*tm502*7) worms had the same percent growth (L1 to adult) as N2 worms, suggesting that background mutation was sufficiently restored (Figure S1).

### 2.3. Synchronizations of Worms

Starved L1 larvae were prepared in order to obtain synchronized worms as previously described [10]. In brief, worms on NGM plates were harvested and incubated in alkaline hypochlorite [500 mM NaOH and 1.2% (*v*/*v*) hypochlorite] until their bodies were completely dissolved (5–10 minutes). Eggs were then washed 3 times with S basal [50 mM potassium phosphate (pH 6.0) and 100 mM NaCl]. Eggs were hatched and synchronized by incubation at 20°C overnight without food.

### 2.4. L1 Growth Assay

The time-course drug treatments were performed using synchronized L1 worms as previously described [10]. The synchronized L1 larvae were treated with several drugs in M9 buffer at 20°C. Then, the worms were transferred to NGM plates and incubated for 4 days. Synchronized L1 worms were irradiated with *γ*-rays and UVC on NGM plates and then incubated for 4 days. After 4 days, the percentage of worms that grew from L1 to adults was calculated. At least 150 animals were counted for each condition.

### 2.5. Adult Worm Drug Resistance Assay

Drug resistance assays using adult worms were performed as previously described [10]. Briefly, synchronized L1 larvae were cultured on NGM plates until they completely developed to the adult stage (4 days). They were harvested and then treated with drugs for 1 hour at 20°C in M9 buffer. They were transferred to NGM plates and incubated for 1 day. The percent survival was then calculated. At least 150 animals were counted for each condition.

### 2.6. AO Staining

AO staining was performed as previously described [10]. Briefly, synchronized L1 larvae were cultured on NGM plates until they developed completely to the adult stage (4 days). Adult worms were harvested and treated with drugs in M9 buffer for 1 hour at 20°C. The worms were then washed with M9 buffer twice and stained with 5 mg/ml acridine orange (AO) in M9 buffer for 5 minutes. The worms were destained twice with 1 ml of M9 buffer for 10 minutes and fixed with phosphate-buffered saline (pH 7.4) (PBS) containing 4% paraformaldehyde (PFA). After washing twice with 1 ml of M9 buffer, they were observed by fluorescence microscopy with excitation by a 488 nm argon laser. At least 150 animals were counted for each condition. The microscopy was performed with a Carl Zeiss LSM510 microscope (Carl Zeiss, Germany).

### 2.7. Reporter Assay

The *Lgg-1* reporter assay was performed as previously described [10]. Briefly, *lgg-1* reporter (*tm5027* [*lgg-1*p::GFP::*lgg-1* + *odr-1*p::RFP]) adult worms were harvested with M9 buffer and treated with drugs for 7 hours at 20°C in M9 buffer. The worms were fixed with PBS containing 4% PFA for 10 minutes at 20°C. After washing twice with M9 buffer, the worms were observed by fluorescence microscopy with excitation by a 488 nm argon laser. At least 150 animals were counted for each condition. The microscopy was performed with a Carl Zeiss LSM510 microscope (Carl Zeiss, Germany).

### 2.8. Statistics

Qualitative data were representative data of at least three experiments. Unless otherwise noted, quantitative data were expressed as the mean ± S.D. The significance of differences was examined by Student's *t*-test. *p* < 0.05 was considered significant.

## 3. Results

### 3.1. *atm-1*(*tm5027*) Worms Were Sensitive to DNA-Damaging Agents

Many studies using cultured mammalian cells have demonstrated that dysfunction of ATM results in sensitivity to ROSs and several DNA-damaging agents, including *γ*-rays [17, 18]. Previous studies using *C. elegans*reported that *atm-1*(*tm502*7) germ cells are sensitive to *γ*-rays and UVC [11, 19]. We also found that *atm-1*(*tm502*7) L1 larvae are sensitive to the SN1-type alkylating agent N-methyl-N′-nitro-N-nitrosoguanidine (MNNG) and SN2-type alkylating agent MMS [10]. However, the sensitivity to MNNG of *amt-1*(*tm5027*) larvae was dependent on mismatch repair (MMR), whereas sensitivity to MMS was not [10]. These differences in sensitivities to both types of alkylating agents suggest that the mechanism of DSB generation is largely dependent on other DNA repair pathways in *C. elegans* somatic cells. Therefore, a comprehensive analysis using different DNA-damaging agents is needed to understand which kinds of DNA damage generate DSBs. Thus, we first performed drug treatment assays using L1 larvae with several DNA-damaging agents.

We treated L1 larvae with *γ*-rays, UVC, and a crosslinking agent (mitomycin C; MMC).

*γ*-rays induce DSBs in genomic DNA by two ways: direct breaking or indirect breaking via generation of ROSs [20]. UVC generates pyrimidine dimers in genomic DNA [21]. As pyrimidine dimers strongly block transcription, they result in DSBs [22, 23]. MMC is an antitumor drug that alkylates genomic DNA and forms interstrand crosslinking (ICL) [24]. Due to the high ability of ICL to block the progression of replication and transcription, the accumulation of ICLs leads to generation of DSBs during replication and transcription [25]. Different types of DNA-damaging agents all generate DSBs in different ways.

**Figure 1:**
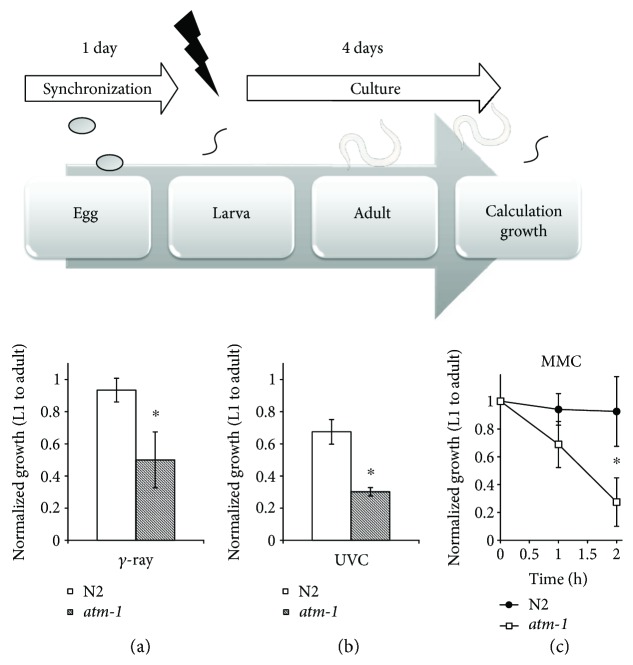
*atm-1*(*tm5027*) worms were sensitive to several DNA-damaging agents. (a–c) Synchronized L1 larvae of N2 (white bar or black diamond) and *atm-1*(*tm5027*) (gray bar and white bar) were irradiated with (a) 100 Gy of *γ*-rays or (b) 15 J/m^2^ UVC light or underwent time-course treatment with (c) 0.2 mg/ml MMC at 20°C. After treatments, the worms were cultured for 4 days and we calculated the ratio of adult worms/transferred L1 worms. All data are mean ± SD and ∗ means significantly different by Student's *t*-test (*p* < 0.05). The photograph of a worm was obtained from TogoTV (© 2016 DBCLS TogoTV).

Using these different DSB sources, we assessed whether they induce DSBs in *C. elegans* dividing somatic cells. As shown in Figures 1(a)–1(c), *atm-1*(*tm5027*) larvae exhibited sensitivity to almost all of the DNA-damaging agents.

Interestingly, *atm-1*(*tm5027*) adult worms were sensitive to NaHSO_3_, a deaminating agent that induces DSBs via formation of uracil in genomic DNA, but *atm-1*(*tm5027*) larvae were not (Figures S2A and B).

### 3.2. *atm-1*(*tm5027*) Adult Worms Exhibited Resistance to H_2_O_2_

**Figure 2:**
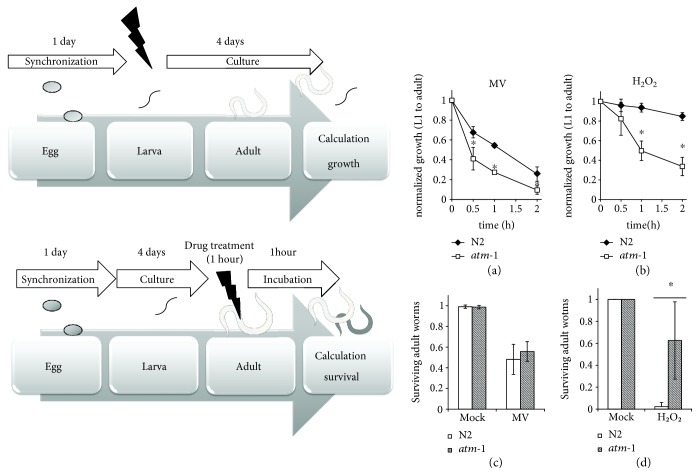
*atm-1*(*tm5027*) larvae exhibited sensitivity, but adult worms were resistant to H_2_O_2_. (a, b) Synchronized L1 larvae of N2 and *atm-1*(*tm5027*) underwent time-course treatment with (a) 40 mM MV or (b) 0.5 mM H_2_O_2_ at 20°C. After treatments, the worms were cultured for 4 days and we calculated the ratio of adult worms/transferred L1 worms. (c, d) Synchronized adult worms of N2 (white bars) and *atm-1*(*tm5027*) (gray bars) were treated with (c) 200 mM MV or (d) 88 mM H_2_O_2_ for 1 hour at 20°C. 24 hours after these treatments, the percent survival was calculated. All data are mean ± SD and ∗ means significantly different by Student's *t*-test (*p* < 0.05). The photograph of a worm was obtained from TogoTV (© 2016 DBCLS TogoTV).

Next, we evaluated the sensitivity of *atm-1*(*tm5027*) worms to ROSs using H_2_O_2_ and MV. H_2_O_2_ is a typical ROS generated in vivo [26] and is used in some signaling pathways [27, 28]. MV generates O_2_^−^ in vivo [29, 30]. First, we tested the sensitivity of larvae, and *atm-1*(*tm5027*) larvae exhibited sensitivity to both H_2_O_2_ and MV (Figures 2(a) and 2(b)).

In general, ROSs also generate DSBs and activate cell cycle checkpoints in dividing cells [31, 32]. Therefore, because of the lack of cell cycle checkpoints, adult worms were expected to have different responses to ROSs from those of larvae. In order to analyze the response to ROSs of CeATM-1, we treated fully matured worms with H_2_O_2_ and MV.

Drug resistance assays using adult worms demonstrated that *atm-1*(*tm5027*) adult worms had significant resistance to H_2_O_2,_ but similar sensitivity to MV as wild-type (Figures 2(c) and 2(d)).

### 3.3. CeATM-1 Induces Intestinal Cell Death in Response to H_2_O_2_ Treatment

**Figure 3:**
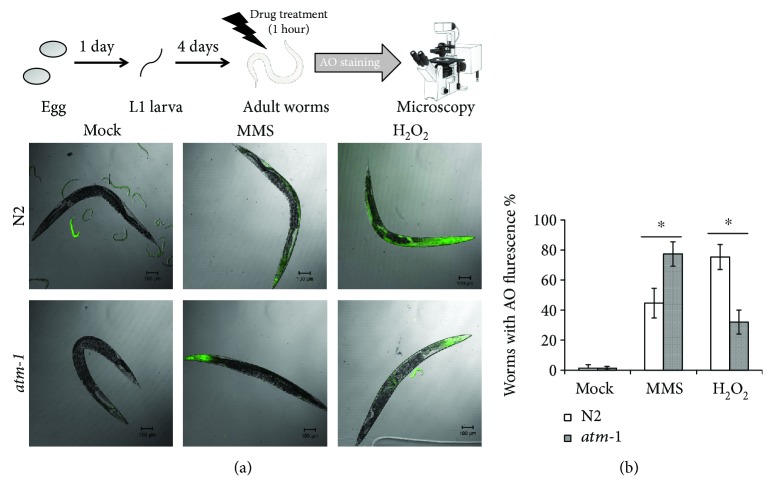
CeATM-1 induces intestinal cell death in response to H_2_O_2_. (a) Adult N2 and *atm-1*(*tm5027*) worms were treated with 0.5% MMS or 88 mM H_2_O_2_ for 1 hour at 20°C. After the treatment, the worms were stained with AO for 5 minutes at 20°C and observed microscopically after destaining twice with M9 buffer and fixing with PFA. (b) The fraction of animals with somatic AO fluorescence. At least 150 animals were counted for each condition. All data are mean ± SD and ∗ means significantly different by Student's *t*-test (*p* < 0.05). The photographs of a worm and a microscope were obtained from TogoTV (© 2016 DBCLS TogoTV).

In order to address the mechanisms of resistance of *atm-1*(*tm5027*) adult worms to H_2_O_2_, we performed dead cell imaging using acridine orange AO [33]. AO is a nonfluorescent dye that fluoresces only when it stably binds to nucleic acids [33]. As AO is actively exported out of the living cells, fluorescence is observed only in dead cells after sufficient destaining [33]. In nontreated worms, fluorescence was not detected in both wild-type and *atm-1*(*tm5027*) worms (Figure 3(a)). The head, which contains the pharynx and neurons, was severely injured by the MMS treatment in *atm-1*(*tm5027*) worms, whereas that of wild-type worms was not (Figures 3(a) and 3(b)). On the other hand, the intestines of wild-type worms were severely injured by the H_2_O_2_ treatment, whereas those of *atm-1*(*tm5027*) worms were not (Figures 3(a) and 3(b)).

**Figure 4:**
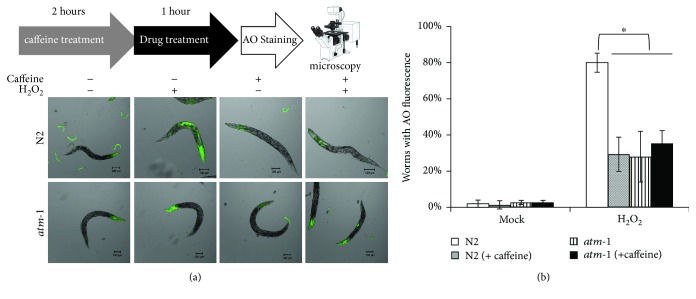
Caffeine prevents H_2_O_2_-dependent intestinal cell death. (a) Adult N2 and *atm-1*(*tm5027*) worms were pretreated with caffeine (20 mM caffeine for at least 6 hours). Then, the worms were treated with 88 mM H_2_O_2_ for 1 hour at 20°C. After the treatment, the worms were stained with AO for 5 minutes at 20°C and observed microscopically after destaining twice with M9 buffer and fixing with PFA. (b) The fraction of animals with somatic AO fluorescence. At least 150 animals were counted for each condition. All data are mean ± SD and ∗ means significantly different by a multiple-comparison one-way ANOVA (Tukey's test) (*p* < 0.05).

For further confirmation, we next observed AO fluorescence under caffeine treatment. Caffeine is often used as an inhibitor of ATM and ATR proteins [34]. In a previous study, 1 mM caffeine decreased ATM kinase activity to approximately 20% [34]. We pretreated adult worms with 2.5 mM caffeine for 2 hours before the H_2_O_2_ treatment and then treated them with 88 mM H_2_O_2_ for 1 hour followed by AO staining. Caffeine at 2.5 mM did not affect wild-type or *atm-1*(*tm5027*) worms without H_2_O_2_ treatment (Figures 4(a) and 4(b)). In contrast, caffeine significantly suppressed the intestinal cell death in wild-type worms, but it did not in *atm-1*(*tm5027*) worms (Figures 4(a) and 4(b)). These results indicate that CeATM-1 induced intestinal cell death in response to H_2_O_2_.

### 3.4. CeATM-1 Induces Cell Death with Autophagy in Response to H_2_O_2_ Treatment

Next, we tried to identify the type of cell death induced by CeATM-1. In mammalian dividing cells, ATM generally induces apoptosis via phosphorylation of p53 [35]. However, in *C. elegans*, CEP-1 (p53 homologue in *C. elegans*) is abundantly expressed in dividing cells like germ cells, but is hardly expressed in nondividing somatic cells [36]. Therefore, we examined p53-independent cell death pathway. Previously, we reported that MMR induces cell death with autophagy in *C. elegans* nondividing somatic cells [10]. In addition, emerging evidence suggests that ATM plays key roles in autophagy, mitophagy, and pexophagy [37, 38]. Thus, we examined whether ATM induces cell death with autophagy in response to H_2_O_2_.

**Figure 5:**
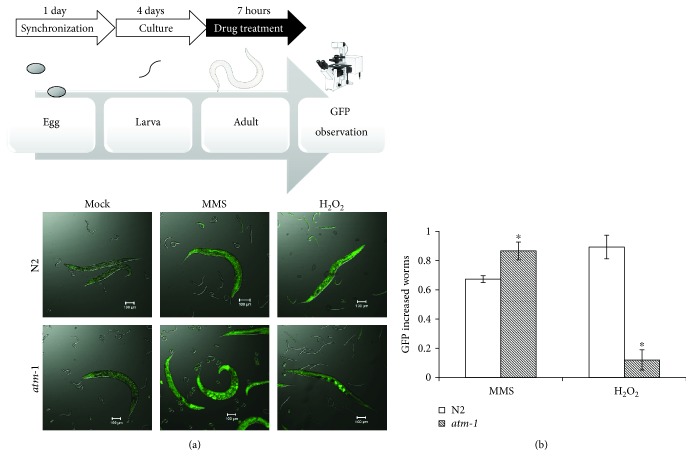
CeATM-1 induces cell death with autophagy in response to H_2_O_2_. (a) The synchronized adult MAH236 (*lgg-1*p::GFP::*lgg-1* + *odr-1*p::RFP) and *atm-1*(*tm5027*/[*lgg-1*p::GFP::*lgg-1* + *odr-1*p::RFP]) worms were pretreated with 20 mM caffeine for 6 hours. Then, the worms were treated with 0.25% MMS or 44 mM H_2_O_2_ for 7 hours at 20°C. After the treatments, the worms were fixed with PFA. After washing twice with M9 buffer, the worms were examined microscopically. (b) The fraction of animals with increased somatic GFP. At least 150 animals were counted for each condition. All data are mean ± SD and ∗ means significantly different by Student's *t*-test (*p* < 0.05). The photographs of a worm and a microscope were obtained from TogoTV (© 2016 DBCLS TogoTV).

In *C. elegans*, the increase of LGG-1 (Atg8/LC3 homologue in *C. elegans*), a member of the autophagosome, is a marker of autophagy [39]. We made an *lgg-1* reporter strain (*tm5027* [*lgg-1*p::GFP::*lgg-1* + *odr-1*p::RFP]) and performed reporter assays. The expression of *lgg-1* was elevated by H_2_O_2_ treatment in wild-type intestinal cells (Figures 5(a) and 5(b)). In contrast, the expression level of *lgg-1* was not increased in *atm-1*(*tm5027*) somatic cells, but increased expression of *lgg-1* was observed in the embryos held in *atm-1*(*tm5027*) adult worms (Figure 5(a)). In addition, we observed expression of *lgg-1* after the MMS treatment. The expression of *lgg-1* was elevated by MMS treatment in both the somatic cells and embryos of *atm-1*(*tm5027*) worms, but its induction was not observed in wild-type worms (Figures 5(a) and 5(b)). These results suggest that H_2_O_2_ and MMS induced cell death with autophagy.

## 4. Discussion

In the present study, we obtained interesting finding that *atm-1*(*tm5027*) adult worms were resistant to H_2_O_2_.

A recent study revealed that ATM is directly activated by H_2_O_2_ and becomes an active dimer [4]. Thus, ATM is considered to be a sensor of ROSs. In addition, ATM is known as a regulator of ROSs [37, 38]. Previous studies reported that ATM downregulates cellular ROS levels via phosphorylation of p53 [40]. Dysfunction of ATM is known to result in diabetes by increasing ROSs followed by abnormal activation of the ASK1/JNK pathway [41]. Therefore, ATM is considered to function as a sensor and direct regulator of ROSs.

As a further role, we found that ATM can induce cell death in response to H_2_O_2_ (Figure 3). H_2_O_2_ ATM dependently induced intestinal cell death. The lack of ATM in adult worms resulted in resistance to H_2_O_2_ (Figure 2(d)). On the other hand, *atm-1*(*tm5027*) larvae exhibited sensitivity to H_2_O_2_ (Figure 2(b)). This difference is considered to be due to the cell cycle checkpoints. ATM plays a central role in DDR, especially in DSB repair [2]. Therefore, dividing cells that have dysfunctional ATM are sensitive to ROSs because ROSs induce DSBs in genomic DNA and activate cell cycle checkpoints [31, 32]. On the other hand, nondividing cells do not have cell cycle checkpoints. This demonstrates the advantage of *C. elegans* for analyzing the function of ATM.

Interestingly, *atm-1*(*tm5027*) adult worms did not exhibit sensitivity to O_2_^−^ (Figure 2(c)). Previously, it was reported that pretreatment of pyocyanin, one of the O_2_^−^-inducing chemicals, inhibits ATM activation induced by *γ*-rays irradiation [42]. As H_2_O_2_ and O_2_^−^ have different oxidation potentials [43], this difference implies that O_2_^−^ cannot induce ATM-1 signaling in response to ROSs due to too strong oxidation potentials, which may result in excessive oxidation of ATM.

Recent studies highlighted the regulation of autophagy by ATM. Alexandera et al. revealed that ATM induces autophagy via the LKB/AMPK/mTOR pathway in human cultured cells [44]. In addition, Qi et al. reported that Parkin accumulates in response to spermidine treatment followed by ATM activation, and mitophagy is activated [45]. In our study, *C. elegans* adult worms exhibited ATM-dependent cell death with autophagy (Figure 5). Our results suggest a new significance of induction of autophagy by ATM. However, cell death with autophagy may be unique to *C. elegans*. Because *C. elegans* adult somatic cells have the unique condition of little caspase or CEP-1, and ATM usually induces apoptosis via p53 in mammalian cells [1, 36], and abnormality of ATM induces ataxia-telangiectasia in humans (3), *atm-1*(*tm5027*) did not have obvious abnormalities in behavior.

Previously, we reported that MMR induces intestinal cell death with autophagy by MV treatment [10]. MMR also induced cell death with autophagy by MNNG treatment, but in the pharynx and neurons [10]. This type of tissue-specific response was also observed in CeATM-1 (Figures 3 and 5). As discussed in the previous report, the *C. elegans* intestine may be more sensitive to ROSs than other tissues, and how those differences are established needs to be elucidated.

In summary, we found that ATM induces cell death in response to H_2_O_2_. In general, cells lacking ATM are thought to be sensitive to ROSs, but our results demonstrated that lack of ATM results in resistance to H_2_O_2_ in nondividing conditions (Figure 2(d)). Thus, the lack of ATM-dependent cell death may be one reason why the cerebellar ataxia is observed in AT patients but not in NBS patients [3]. We are considering that the lacks of these ATM-dependent cell deaths might be the one of the reasons of the cerebellar ataxia. However, why do the resistant AT patient's cerebellar cells are dysfunctional is unknown. We hypothesize that ATM prevents necrosis, which injures other cells via release of lysosomal enzymes, by inducing cell deaths with autophagy in response to H_2_O_2_, protecting the tissue integrity.

## 5. Conclusion

In this study, we found that deficiency of ATM results in tolerance to H_2_O_2_ in nondividing cells. We demonstrated that ATM can induce cell death in response to H_2_O_2_, but not O_2_^−^, and this cell death is not apoptosis but cell death with autophagy.

## Data Availability

All data used to support the findings of this study are included within the article.

## Supplementary Materials

Supplementary Materials Supplementary Table: the primers used in this study. These primers were used for the verification of the atm-1(tm5027) deletion allele. Supplementary Figure S1: percent growth of atm-1(tm5027) worms. The percent growth (L1 to L4). Data represent the mean ± S.D. from five independent experiments. N.S. means not significantly different (*p* value ≥ 0.05 by Student's *t*-test). Supplementary Figure S2: drug resistance assay for NaHSO3. (A) Synchronized L1 larvae of N2 (◆) and atm-1(tm5027) (□) were treated with 50 mM NaHSO3 at 20°C. After treatments, the worms were cultured for 4 days and we calculated the ratio of adult worms/transferred L1 worms. (B) Synchronized adult N2 (white bars) and atm-1(tm5027) (gray bars) worms were treated with 105 mM NaHSO3 for 1 hour at 20°C. 24 hours later, the percent survival was calculated. All data are mean ± SD and ∗ means significantly different by Student's *t*-test (*p* < 0.05).
