# Supplementary material for: ATM Induces Cell Death with Autophagy in Response to H2O2 Specifically in Caenorhabditis elegans Nondividing Cells
Source: Oxid Med Cell Longev. 2018 Jul 2;2018:3862070. doi: 10.1155/2018/3862070 (PMC6051064; doi:10.1155/2018/3862070)
Supplement: Supplementary Materials — Supplementary Table: the primers used in this study. These primers were used for the verification of the atm-1(tm5027) deletion allele. Supplementary Figure S1: percent growth of atm-1(tm5027) worms. The percent growth (L1 to L4). Data represent the mean ± S.D. from five independent experiments. N.S. means not significantly different (p value ≥ 0.05 by Student's t-test). Supplementary Figure S2: drug resistance assay for NaHSO3. (A) Synchronized L1 larvae of N2 (◆) and atm-1(tm5027) (□) were treated with 50 mM NaHSO3 at 20°C. After treatments, the worms were cultured for 4 days and we calculated the ratio of adult worms/transferred L1 worms. (B) Synchronized adult N2 (white bars) and atm-1(tm5027) (gray bars) worms were treated with 105 mM NaHSO3 for 1 hour at 20°C. 24 hours later, the percent survival was calculated. All data are mean ± SD and ∗ means significantly different by Student's t-test (p < 0.05). [file 3862070.f1.pdf]

| | | |
|---|---|---|
| *atm-1* (External) | Forward | 5'-CGGAAAAACGATGTACCGATGGCCA-3' |
| | Reverse | 5'-AGGGACCTGCGTCTCTCTTCGCCAC-3' |
| *atm-1* (Internal) | Forward | 5'-CGGAAAAACGATGTACCGATGGCCA-3' |
| | Reverse | 5'-CCGAAGCATCGCCTTCTCCAACATA-3' |

**Supplementary Table.** The primers used in this study. These primers were used for the verification of the *atm-1*(*tm5027*) deletion allele.

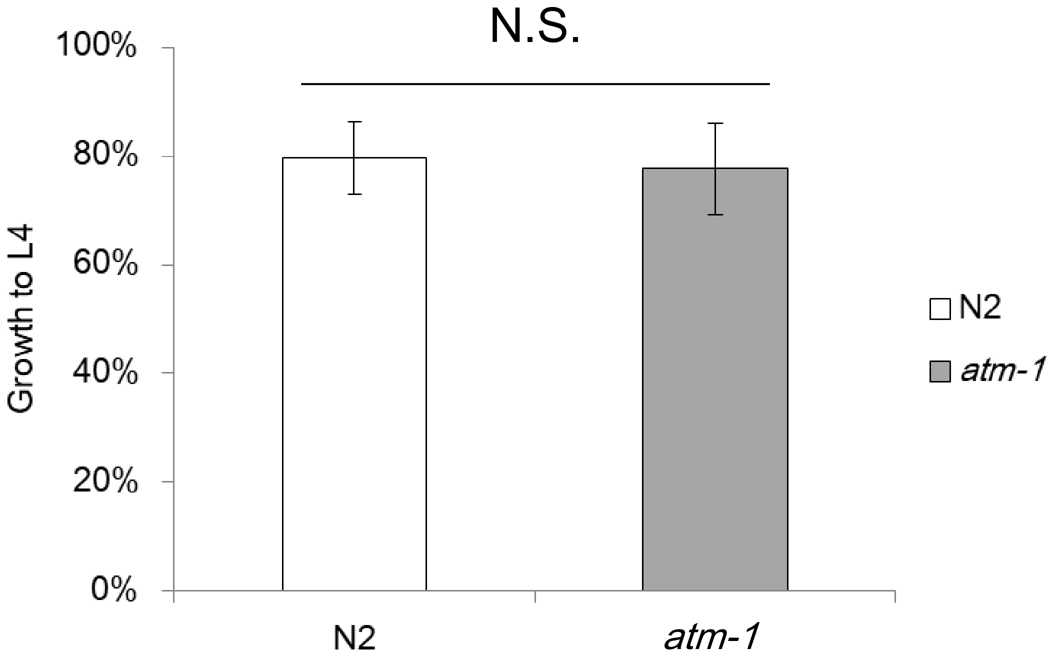


**Supplementary Figure S1. Percent growth of *atm-1*(*tm5027*) worms.** The percent growth (L1 to L4). Data represent the mean ± S.D. from five independent experiments. N.S. means not significantly different (*p*-value ≥ 0.05 by Student's *t*-test).

Fig. S1

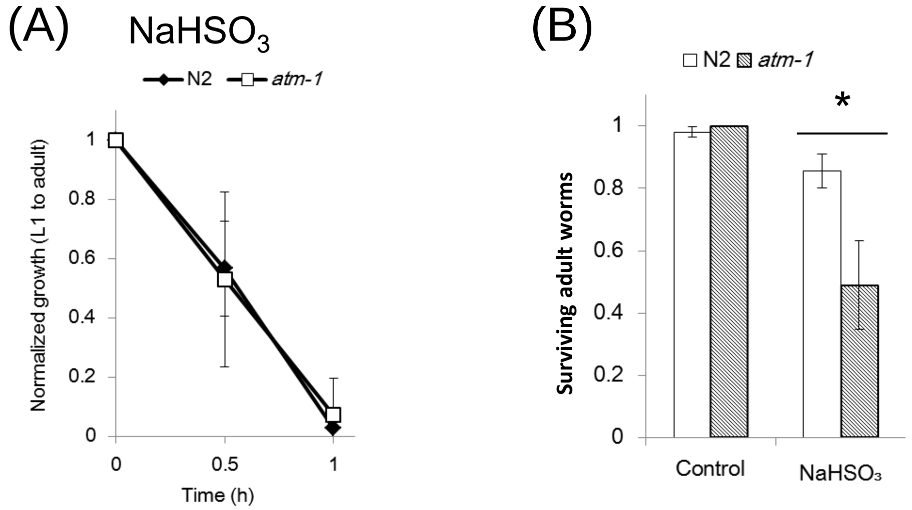


**Supplementary Figure S2. Drug resistance assay for $NaHSO_3$.** (A) Synchronized L1 larvae of N2 (◆) and *atm-1*(*tm5027*) (☐) were treated with 50 mM $NaHSO_3$ at 20$^o$C. After treatments, worms were cultured for 4 days and we calculated the ratio of adult worms / transferred L1 worms. (B) Synchronized adult N2 (white bars) and atm-1(tm5027) (gray bars) worms were treated with 105 mM $NaHSO_3$ for 1 hour at 20$^o$C. 24 hours later, the percent survival was calculated. All data are mean ± SD and * means significantly different by the Student's *t*-test ($p$ < 0.05).

Fig. S2
